# Strain Amplification Analysis of an Osteocyte under Static and Cyclic Loading: A Finite Element Study

**DOI:** 10.1155/2015/376474

**Published:** 2015-01-15

**Authors:** Liping Wang, Jianghui Dong, Cory J. Xian

**Affiliations:** ^1^Sansom Institute for Health Research, School of Pharmacy and Medical Sciences, University of South Australia, Adelaide, SA 5001, Australia; ^2^College of Mechanical and Electronic Engineering, Shanghai Jianqiao University, Shanghai 201319, China; ^3^School of Computer Science, Engineering and Mathematics, Flinders University, Adelaide, SA 5042, Australia

## Abstract

Osteocytes, the major type of bone cells which reside in their lacunar and canalicular system within the bone matrix, function as biomechanosensors and biomechanotransducers of the bone. Although biomechanical behaviour of the osteocyte-lacunar-canalicular system has been investigated in previous studies mostly using computational 2-dimensional (2D) geometric models, only a few studies have used the 3-dimensional (3D) finite element (FE) model. In the current study, a 3D FE model was used to predict the responses of strain distributions of osteocyte-lacunar-canalicular system analyzed under static and cyclic loads. The strain amplification factor was calculated for all simulations. Effects on the strain of the osteocyte system were investigated under 500, 1500, 2000, and 3000 microstrain loading magnitudes and 1, 5, 10, 40, and 100 Hz loading frequencies. The maximum strain was found to change with loading magnitude and frequency. It was observed that maximum strain under 3000-microstrain loading was higher than those under 500, 1500, and 2000 microstrains. When the loading strain reached the maximum magnitude, the strain amplification factor of 100 Hz was higher than those of the other frequencies. Data from this 3D FE model study suggests that the strain amplification factor of the osteocyte-lacunar-canalicular system increases with loading frequency and loading strain increasing.

## 1. Introduction

While bone-forming cells osteoblasts and resorbing cells osteoclasts make up only small proportions (<5 and <1%, resp.), osteocytes make up ~90–95% of all bone cells in adult skeletons [1, 2]. Osteocytes are the longest living bone cells, which can live up to decades within their mineralized environment and have the potential to live as long as the organism itself [3]. The osteocytes are believed to be the cells that sense the mechanical loads of the bone. The major known function of osteocytes is to translate the mechanical strain signals into biochemical signals between osteocytes and cells in the bone [4–6]. Osteocytes are embedded within the bone matrix, can remodel the perilacunar matrix (PCM), and are connected to each other by slender cell processes located within the small bony tubes of the canaliculi [1, 4, 7].

**Figure 1:**
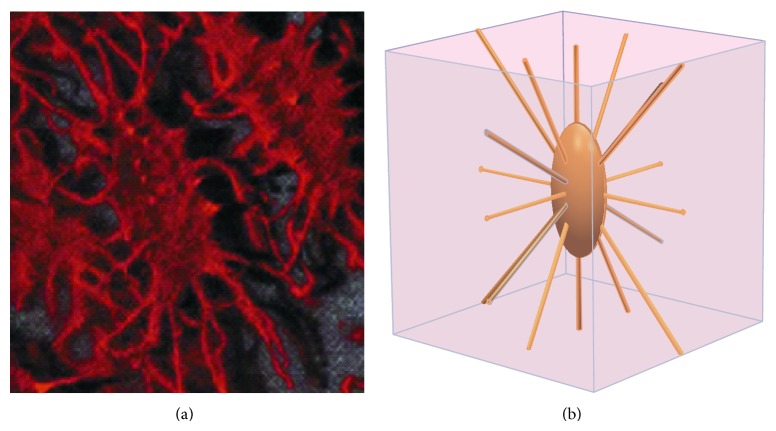
The osteocyte-lacunar-canalicular system. (a) Photomicrograph of osteocytes embedded in bone matrix (adopted from [34]); (b) schematic geometry of cubic volume of bone surrounding one osteocyte lacuna with canaliculi and processes.

Osteocytes are surrounded by the PCM, which is a thin hyaluronan-rich coating. The PCM plays an important role in many cell-surface phenomena; it transfers mechanical signals between the cell body and cell processes and between PCM and the surrounding extracellular matrix (ECM) [8–13]. The PCM represents the entire space between the ECM and the osteocyte cell [14]. Due to these functional relations, the osteocyte-lacunar-canalicular system should be investigated based on the cell-PCM-ECM geometric structure (Figure 1).

With the known biomechanosensory function of the osteocyte-lacunar-canalicular system in bone, dynamic mechanical stimulus like cyclic loading has been frequently used for the microscale environment biomechanics investigation [15]. Hsieh and Turner studied the anabolic effect of mechanical loading on bone tissue by modulating the loading frequency in the range of 1–10 Hz [16]. Gururaja et al. developed a 2D poroelastic model to investigate load-induced fluid flow in the lacunar-canalicular system of bone subjected to harmonic bending excitations in the range of 1–10^6^ Hz [17]. Wang et al. investigated the net tracer transport in bone via the lacunar-canalicular porosity by using a mathematical model during cyclic mechanical loading at loading frequency 0.5–100 Hz [18].

To date, some researchers have investigated the osteocyte-lacunar-canalicular system by experimental and computational models [14, 17, 19–22], and the unit of microstrain has been widely used in the loading and data analyses in the cell modelling. McCreadie and Hollister found the strain levels in and surrounding osteocytes were 1700 to 2700 microstrains [21]. Burr et al. measured the strain by surgically implanted strain gages and found that the strain was below 2000 microstrains even under conditions of strenuous activity [23]. Verbruggen et al. conducted a computational investigation of the strain amplification of osteocytes in vivo in response to mechanical stimulation under vigorous physiological activity (3000 *με* loading levels) [14]. In addition, researchers have introduced methodologies to reconstruct biorealistic osteocyte models. A number of methods have been used to study 3D reconstruction of the osteocyte-lacunar-canalicular system. The methods include scanning electron microscopy (SEM) [24–27], serial focused ion beam/scanning electron microscopy (FIB/SEM) [28], transmission electron microscopy (TEM) [29, 30], ultra high voltage electron microscopes (UHVEM) [31], light microscopy (LM) [32, 33], confocal laser scanning microscopy (CLSM) [14, 34–37], atomic force microscopy (AFM) [38–40], computed tomographic (CT) scanning (such as nano-CT and synchrotron radiation micro-CT) [36, 37, 41, 42], and X-ray (like X-ray phase nanotomography and X-ray microscope (TXM)) [43–45]. However, only a few of these models were used in finite element (FE) analysis of the osteocyte-lacunar-canalicular system. Verbruggen and his coworkers developed FE models of lacunar-canalicular network using confocal microscope imaging in rats [14, 46].

While most previous computational geometries were 2D models, several researchers have proposed 3D models (including biorealistic and idealized FE models) for the computational investigation [14, 21, 22, 47–49]. For example, a 3D poroelastic FE model of rat tibia was developed to study the mechanical loads in modulating local flow distributions and concentration gradients within bone tissue. However, due to the simplicity of the geometry, only a thin layer of elements on the endosteal and periosteal surfaces was used in defining the boundary conditions [49]. As results of FE studies depend on many conditions (e.g., geometry, mesh density, element type and order, and material properties), more credible results can be obtained if more accurate geometries are used in the 3D models, when the other parameters have been confirmed.

Thus, although the osteocyte-lacunar-canalicular system has been investigated through the pure experimental tool, mathematics theory, or 2D FE models in previous studies, only a few researchers have used the 3D FE model. In the current study, a 3D FE model was proposed to predict the biomechanical behaviour of the osteocyte-lacunar-canalicular system. Firstly, a 3D geometry of an osteocyte-lacunar-canalicular system incorporating cell body, PCM, and ECM, which is similar to the real dimension, was developed. Secondly, the biomechanical behaviour of the system was investigated by using the 3D model subjected to the static and cyclic loading of different frequencies and magnitudes.

## 2. Material and Methods

### 2.1. Geometric Modelling and Meshing

**Figure 2:**
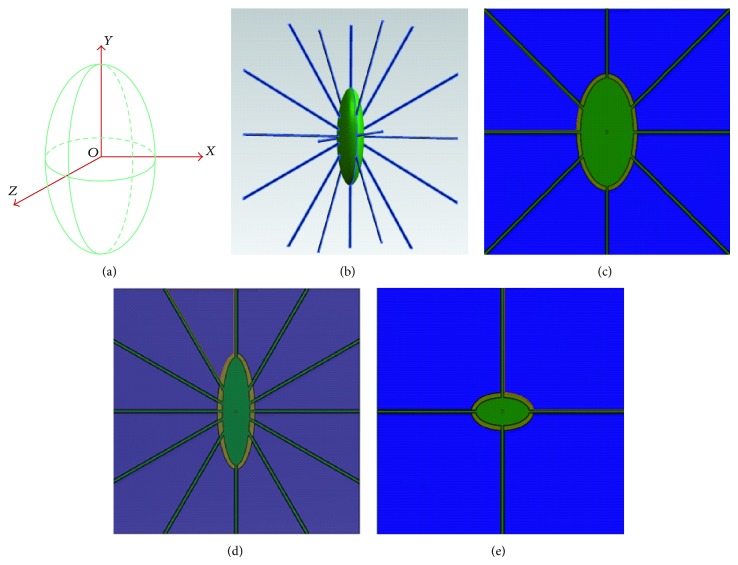
(a) The schematic diagram of an osteocyte lacuna, where *Z* is the intermediate axis, *Y* the major axis, and *X* the minor axis of the lacuna in the local coordinate system. Osteocyte lacuna and canaliculi are shown schematically: (b) osteocyte cell body (green) with processes (blue), (c) *X*-*Y* plane, (d) *Z*-*Y* plane, and (e) *X*-*Z* plane.

An idealized model of osteocyte-lacunar-canalicular system was developed (Figure 2). The triaxial lacunar osteocyte ellipsoid with the Cartesian equation for a general ellipsoid is shown as [50]

(1) $$\begin{array}{c} {\left(\frac{x}{l}\right)}^{2}+{\left(\frac{y}{m}\right)}^{2}+{\left(\frac{z}{n}\right)}^{2}=1, \end{array}$$

where *l*, *m*, and *n* are the osteocyte lacunar semiaxes in the *X*, *Y*, and *Z* direction, respectively. *X* is the minor axis, *Y* the major axis, and *Z* the intermediate axis of the osteocyte lacuna in the local coordinate system. In this study, *l*, *m*, and *n* are 4.6, 9.45, and 2.4, respectively, with the values obtained from human femurs [42]. In this system, the PCM and osteocyte are embedded in the ECM block, whilst the osteocyte is in the center of the ECM block. While the mean length of each direction of ECM is 43 *μ*m for human [50], osteocyte cell bodies are surrounded by a pronounced ~0.5–1 *μ*m thick layer of PCM [51]. The PCM thickness was assumed to be 0.75 *μ*m in this study. Beno et al. [50] estimated the numbers of canaliculi emanating from osteocyte lacunae for different species (six species: chick, rabbit, bovine, horse, dog, and human) by using the slicing method and surface area method based on the data from Remaggi et al. and Ferretti et al. [52, 53]. Canaliculi were idealized as straight cylindrical channels and eighteen canaliculi were modelled as channels in the ECM [50] and using diameter of 0.25 *μ*m [4, 54]. Furthermore, the average width of the canaliculi around the osteocyte process was ~80 nm [4, 55].

**Figure 3:**
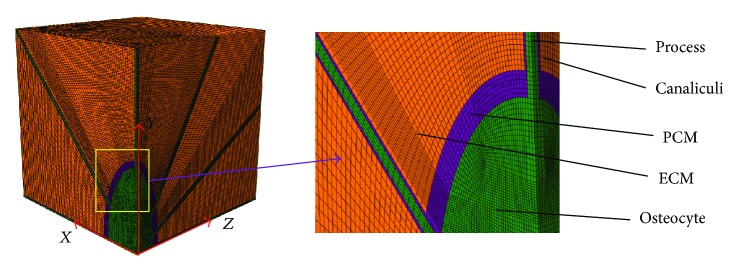
One-eighth symmetry FE mesh model includes ECM, PCM with canaliculi, and osteocyte cell body with processes.

The FE analysis software ABAQUS 6.12 (SIMULIA, Providence, RI, USA) was used for simulations assuming fully saturated media. Because the model is symmetrical, only 1/8 symmetry model was used in all the simulations (Figure 3). The reduced integration first order solid elements in Abaqus suffered from hourglassing when a mesh was coarse. In addition, the fully integration first order solid elements exhibited shear locking. To avoid the elements suffering from hourglassing and shear locking, the reduced integration with second order solid elements is recommended [56]. Twenty-node hexahedral reduced integrated elements (C3D20R) were used for all regions. The hexahedral FE mesh was mapped according to the high quality mesh geometries using twenty-node C3D20R elements in the whole FE analysis. Total number of nodes and elements were 453199 and 107868, respectively, in the 1/8 symmetry model (Table 1). Tie contact interfaces were used to ensure the PCM attached to the ECM and osteocyte and to prevent any relative movement during the simulation.

### 2.2. Materials

All materials were assumed to be isotropic and linearly elastic. The PCM material properties are difficult to measure directly because of its size and connectivity to both cell body and ECM [10]. Meanwhile, it is hard to use the experimental data to define the material properties of the PCM surrounding the osteocyte [14]. An inverse FE approach was used to calculate the linear elastic properties of the PCM. The obtained PCM moduli ranged from 43 to 240 kPa based on this method [10]. Therefore, an elastic modulus of 43 kPa was assigned to the PCM and with Poisson's ratio of 0.4 [9, 14, 57]. The properties of the cells in the lacuna space were assigned a Young's modulus of 10 MPa and Poisson's ratio of 0.4 [21]. A modulus of 4471 ± 198 Pa and Poisson's ratio of 0.3 were attributed to the osteocyte cell body and processes [58]. ECM Young's modulus of 16 GPa and Poisson's ratio of 0.38 were applied. Young's modulus of 43 kPa and Poisson's ratio of 0.4 were used in PCM with canaliculi, and Young's modulus of 4.47 kPa and Poisson's ratio of 0.3 were used for osteocyte cell body and processes. The material was modelled as linear elastic isotropic (Table 1).

### 2.3. Boundary and Loading Conditions

To simulate the mechanical environment of the lacunocanalicular system, static and cyclic loads were applied and the responses of strain and stress distributions were analyzed. The ramped static compressive loads of 500, 1500, 2000, and 3000 microstrain were used in this study [14, 22]. Generally, for most animals, the peak principal compressive strains are about 2000 to 3000 microstrains. However, peak principal strain in the human tibia at the location measured is slightly less than these values. In some cases such as uphill and downhill zigzag running, the principal compressive and shear strains are the greatest, reaching nearly 2000 microstrains, about three times higher than that recorded during walking [23].

**Figure 4:**
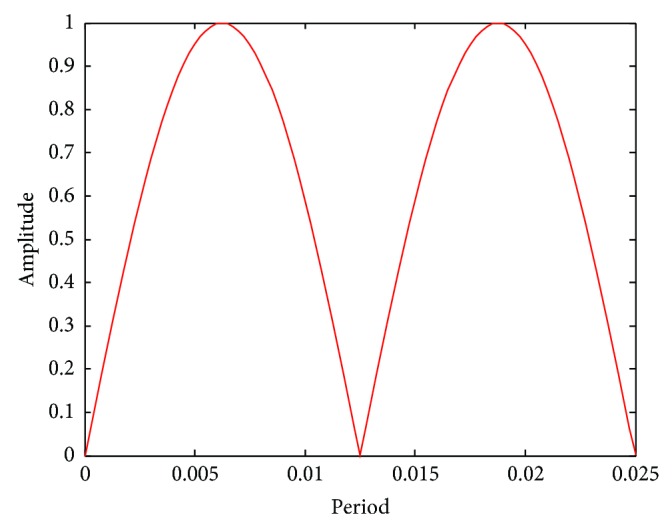
Effects of load cycle on the model.

Furthermore, a sinusoidal loading *A* = |*A*_0_[sin(*ωt*)]| was used for compression [59] (Figure 4). The maximal loading magnitudes *A*_0_ were 500, 1500, 2000, and 3000 microstrains, respectively, in the simulations. The axial displacement boundary conditions were applied to the model faces to simulate the compression. The nodes on the opposing faces of applied displacement loading were constrained symmetrically to prevent rigid body motion [14, 22, 23]. To examine the influence of frequencies on strain, the loading frequencies of 1, 5, 10, 40, and 100 were investigated [16, 17, 59, 60].

## 3. Results 

### 3.1. Static Loading

**Figure 5:**
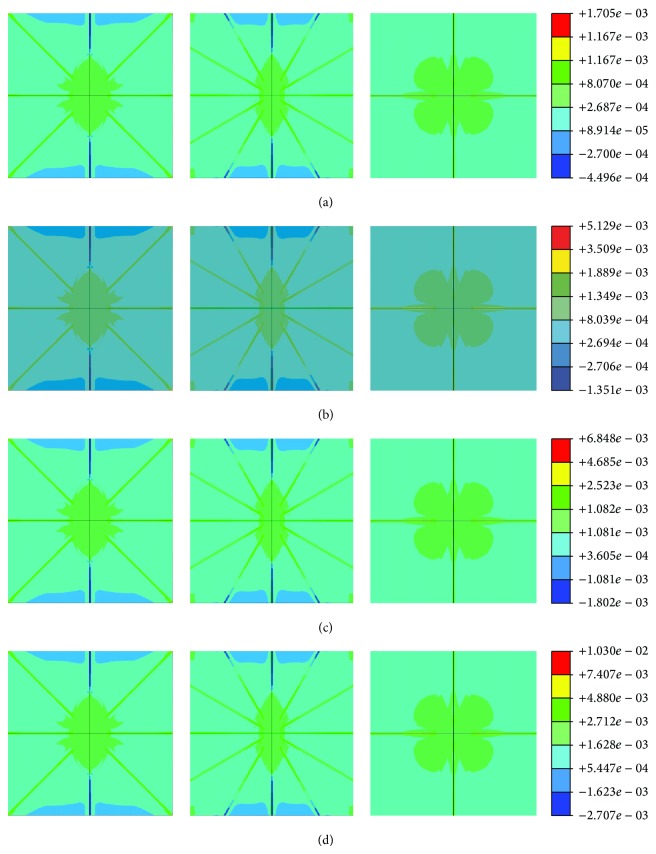
Strain distribution of half FE model in *X*-*Y* plane (left), *Y*-*Z* plane (middle), and *X*-*Z* plane (right), respectively, under different global loading, (a) 500-microstrain global loading, (b) 1500-microstrain global loading, (c) 2000-microstrain global loading, and (d) 3000-microstrain global loading.

To simulate the mechanical environment of the lacunocanalicular system, the global static compressive loads of 500, 1500, 2000, and 3000 microstrains were applied and the responses of strain and stress distributions were analyzed. The strain amplification factor was computed as the local maximum strain divided by the applied global strain as described [9, 22]. The contour plots of half symmetry FE model (Figures 5(a)–5(d)) show the predicted strain distribution of the lacunar-canalicular FE model under 500, 1500, 2000, and 3000 microstrains, respectively, and the maximum principal strains were about 1705, 5129, 6848, and 10300 microstrains, respectively. When subjected to a nominal continuum strain level approximately equal to that measured in humans in vivo during rigorous activity like 2,000 microstrains, the osteocyte level strains can be as high as 12,000 to 15,000 microstrains (1.2% to 1.5%) [61]. Obviously, the maximum principal strain is located in the canaliculi near to osteocyte cell body and the interface between canaliculi and osteocyte. For the osteocyte cell, the principal strains in different areas followed the following trend: innermost center > center > outermost section. For PCM, the principal strains in the top and bottom fractions near to osteocyte cell body were higher than the rest, whilst those in the left and right side sections were lower than the other parts in the ECM.

**Figure 6:**
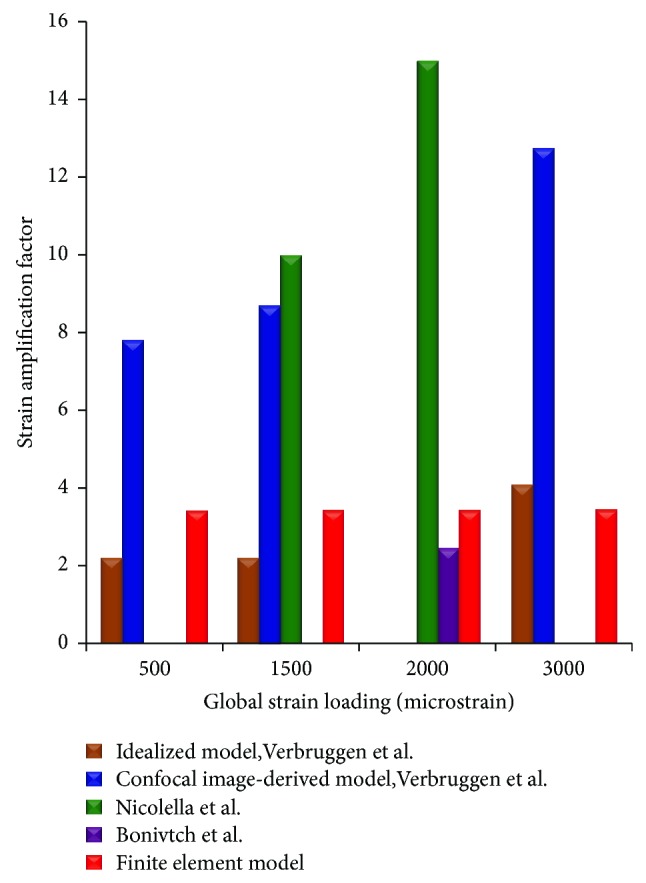
The strain amplification factor of FE mesh model compared with reference data from the literature.

The predicted results of strain amplification factors of the FE model were calculated for different loads (Figure 6), which were compared with the data in the literature [14, 22]. The strain amplification factors were 3.41, 3.419, 3.24, and 3.43 for 500, 1500, 2000, and 3000 microstrain global loads, respectively. The strain amplification factors for loads of 500 and 3000 microstrains were comparable with the values of the idealized model and confocal image-derived model from Verbruggen et al. [14], while that for the loading of 1500 microstrains was comparable with the values of the idealized model from Verbruggen et al. [14] and Nicolella et al. [62]. Finally, the strain amplification factor for 2000-microstrain loading was comparable with those from the idealized model of Rath Bonivtch et al. [22] and Nicolella et al. [62].

### 3.2. Cyclic Loading

**Figure 7:**
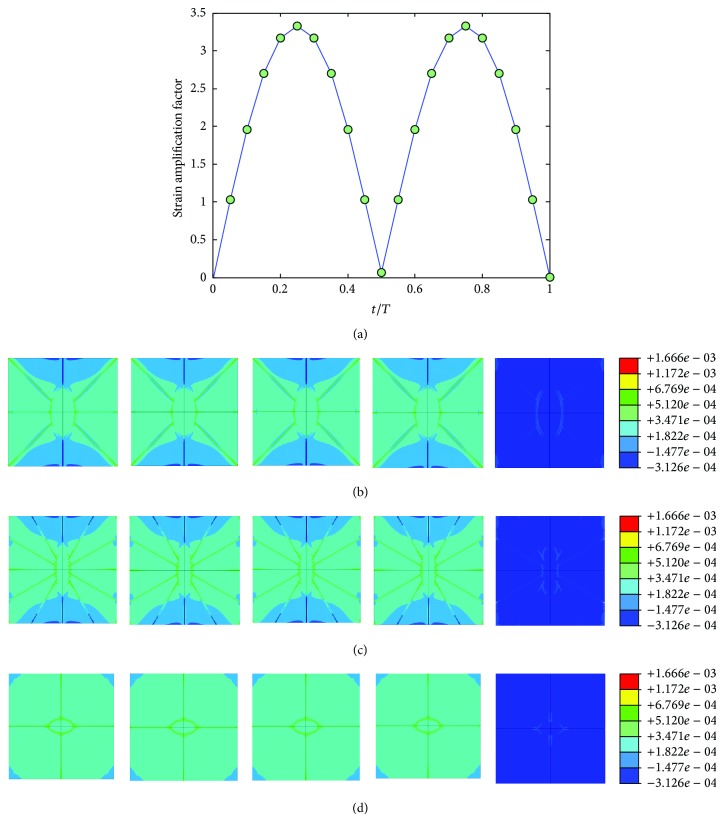
Strain amplification factors and strain distribution by application of a sinusoidal global strain load of 500 microstrains at the frequency of 1 Hz, (a) strain amplification factors versus one loading period, (b) when *t* = 0.1*T*, 0.25*T*, 0.5*T*, 0.75*T*, and 1*T*, strain distribution in *X*-*Y* plane, (c) when *t* = 0.1*T*, 0.25*T*, 0.5*T*, 0.75*T*, and 1*T*, strain distribution in *Y*-*Z* plane, and (d) when *t* = 0.1*T*, 0.25*T*, 0.5*T*, 0.75*T*, and 1*T*, strain distribution in *X*-*Z* plane.

**Figure 8:**
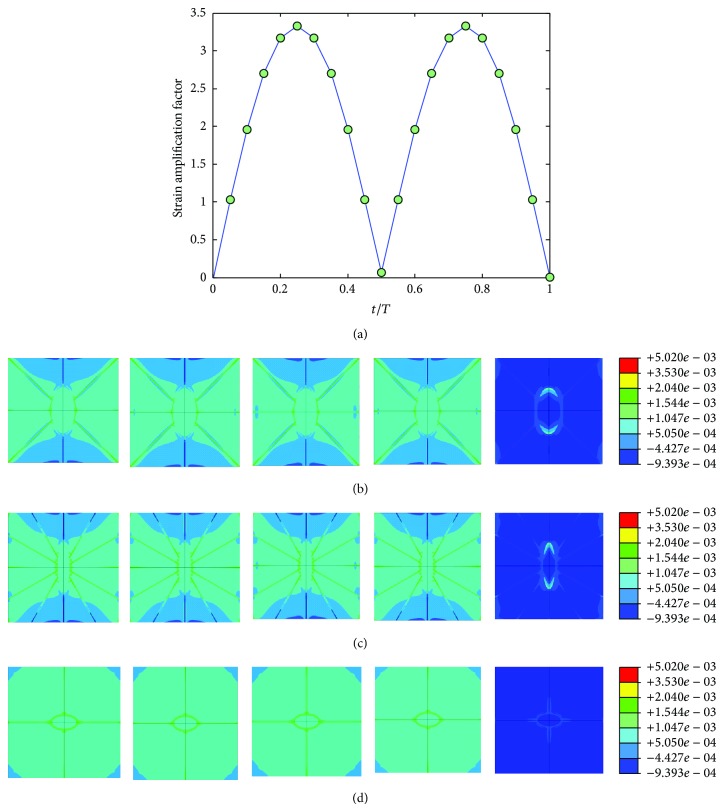
Strain amplification factors and strain distribution by application of a sinusoidal global strain load of 1500 microstrains at the frequency of 5 Hz, (a) strain amplification factors versus one loading period, (b) when *t* = 0.1*T*, 0.25*T*, 0.5*T*, 0.75*T*, and 1*T*, strain distribution in *X*-*Y* plane, (c) when t = 0.1*T*, 0.25*T*, 0.5*T*, 0.75*T*, and 1*T*, strain distribution in *Y*-*Z* plane, and (d) when *t* = 0.1*T*, 0.25*T*, 0.5*T*, 0.75*T*, and 1*T*, strain distribution in *X*-*Z* plane.

**Figure 9:**
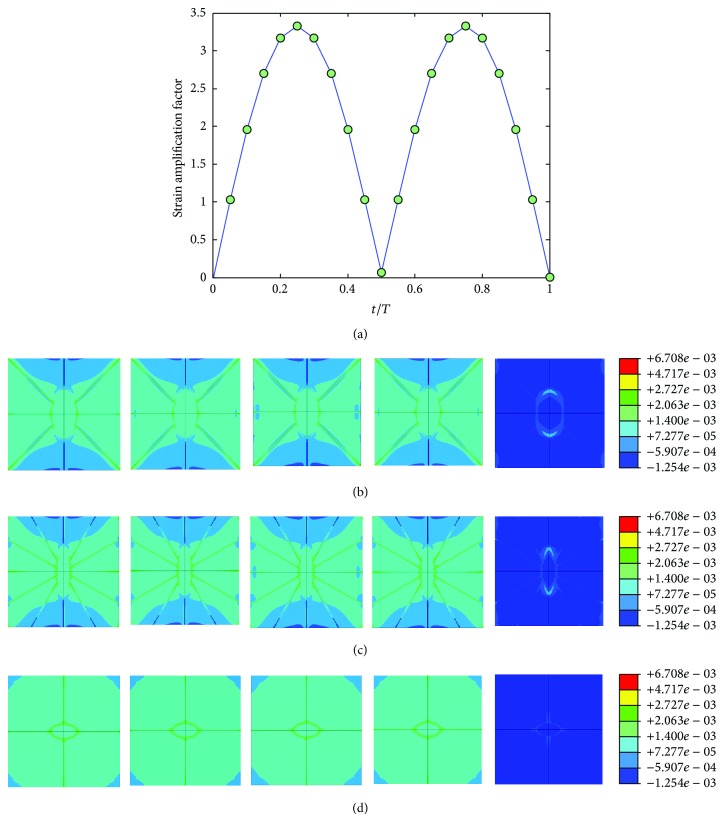
Strain amplification factors and strain distribution by application of a sinusoidal global strain load of 2000 microstrains at the frequency of 10 Hz, (a) strain amplification factors versus one loading period, (b) when *t* = 0.1*T*, 0.25*T*, 0.5*T*, 0.75*T*, and 1*T*, strain distribution in *X*-*Y* plane, (c) when *t* = 0.1*T*, 0.25*T*, 0.5*T*, 0.75*T*, and 1*T*, strain distribution in *Y*-*Z* plane, and (d) when *t* = 0.1*T*, 0.25*T*, 0.5*T*, 0.75*T*, and 1*T*, strain distribution in *X*-*Z* plane.

**Figure 10:**
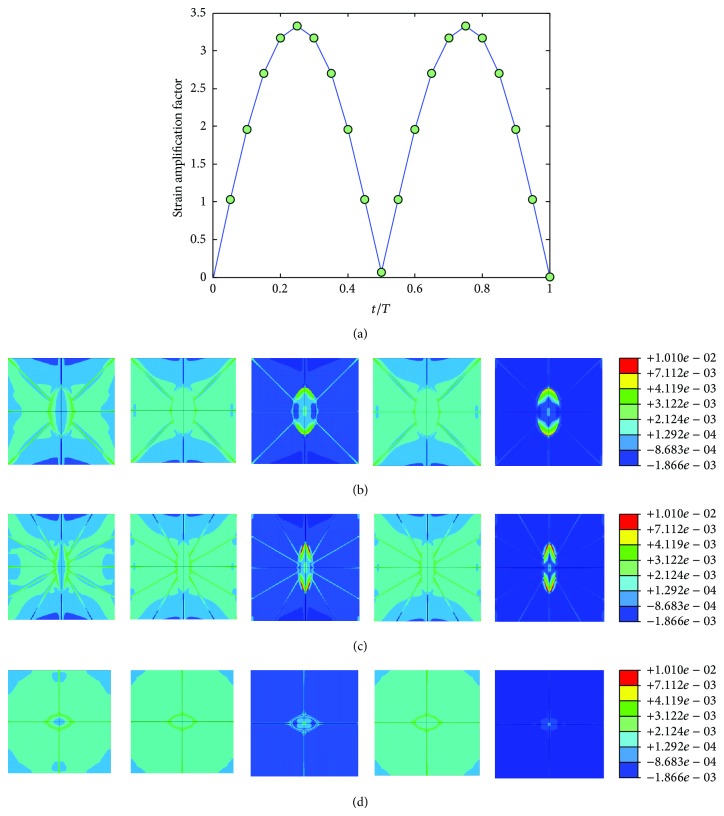
Strain amplification factors and strain distribution by application of a sinusoidal global strain load of 3000 microstrains at the frequency of 40 Hz, (a) strain amplification factors versus one loading period, (b) when *t* = 0.1*T*, 0.25*T*, 0.5*T*, 0.75*T*, and 1*T*, strain distribution in *X*-*Y* plane, (c) when *t* = 0.1*T*, 0.25*T*, 0.5*T*, 0.75*T*, and 1*T*, strain distribution in *Y*-*Z* plane, and (d) when *t* = 0.1*T*, 0.25*T*, 0.5*T*, 0.75*T*, and 1*T*, strain distribution in *X*-*Z* plane.

**Figure 11:**
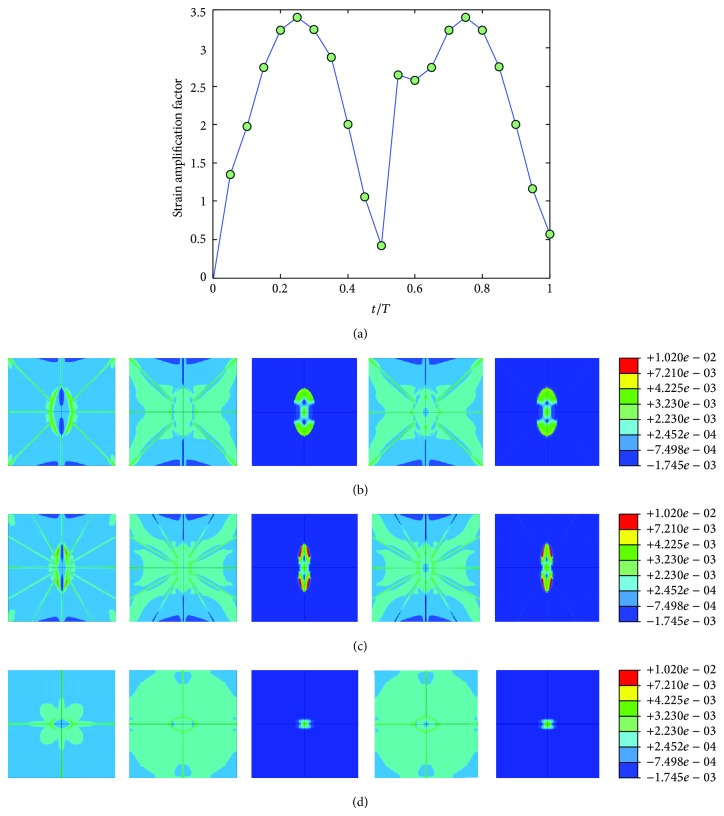
Strain amplification factors and strain distribution by application of a sinusoidal global strain load of 3000 microstrains at the frequency of 100 Hz, (a) strain amplification factors versus one loading period, (b) when *t* = 0.1*T*, 0.25*T*, 0.5*T*, 0.75*T*, and 1*T*, strain distribution in *X*-*Y* plane, (c) when *t* = 0.1*T*, 0.25*T*, 0.5*T*, 0.75*T*, and 1*T*, strain distribution in *Y*-*Z* plane, and (d) when *t* = 0.1*T*, 0.25*T*, 0.5*T*, 0.75*T*, and 1*T*, strain distribution in *X*-*Z* plane.

The mechanical environment response simulations of the osteocyte-lacunocanalicular system were also investigated under cyclic loads with the loading frequencies of 1, 5, 10, 40, and 100 Hz and the loads of 500, 1500, 2000, 3000, and 3000 microstrains. The results of strains were normalized at time increments of *t* = 0.05*T*, where *T* is the relevant period of loading. Figures 7, 8, 9, 10, and 11 show curves of strain amplification factors versus *t*/*T*  (*t*/*T* = 0,0.05,…, 1) and strain distribution (*t*/*T* = 0.1; 0.25; 0.5; 0.75; 1) under loading frequencies of 1, 5, 10, 40, and 100 Hz and strain loads of 500, 1500, 2000, 3000, and 3000 microstrain, respectively.

**Figure 12:**
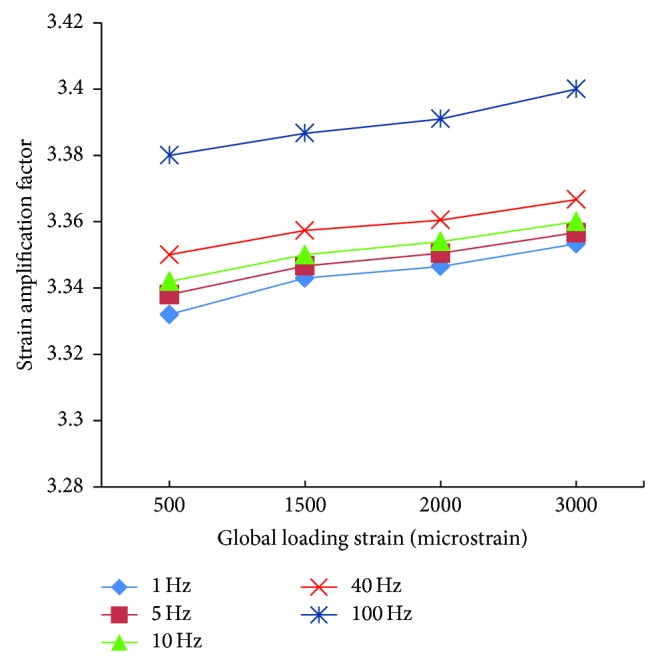
Comparison of strain amplification factors at different frequencies and loading strains all at *t* = 0.5*T*.

The influence of loading frequency and loading magnitude on strain amplification was investigated, and the trends of strain amplification factors at different frequencies (1, 5, 10, 40, and 100 Hz) and loading strains (500, 1500, 2000, and 3000 microstrains) were shown in Figure 12 for *t*/*T* = 0.5.

## 4. Discussion

In this work, a 3D FE model of osteocyte-lacunar-canalicular was developed to investigate the effects of cyclic loading at different frequencies on strain responses of the osteocyte-lacunar-canalicular system. The compressive loads of 500, 1500, 2000, and 3000 microstrains were applied in both static simulation and cyclic simulation. The strain amplification factors of all simulations were also calculated.

Some previous computational studies have examined idealized models of the osteocyte mechanical environment in 3D [21, 22] and have studied both idealized models and biorealistic models [14, 46]; these previous studies represented the first development of accurate 3D FE geometries of the lacuna-osteocyte-canaliculus-ECM system to predict osteocyte mechanobiology. In the previous studies, the shapes of idealized lacunar osteocyte were modelled as revolved ellipsoid with the major and minor axes for idealized models. However, in our study, one triaxial lacunar osteocyte ellipsoid FE model was developed to predict the stain of osteocyte-lacunar-canalicular system for human.

In the static simulation, the strain amplification factors were compared with the existing data in literature (Figure 6). The results of the simulation were found to be in good agreement with the results in literature [14, 22, 62]. The strain amplification factors of simulation were ~3.41, 3.419, 3.424, and 3.43 under 500, 1500, 2000, and 3000 microstrains, respectively, whilst Verbruggen et al. found that these values were 2.2, 2.2, and 4.2 and 7.8, 8.7, and 23.3 for idealized model and confocal image-derived model, respectively [14]. In addition, the strain amplification factor of simulation was about 3.58 under 2000 microstrains, and the result of Rath Bonivtch et al. was 2.45 for idealized model [22]. Compared with the average strain amplification factors (varying from 1.1 to 3.8) from another study [62], the results of our FE analysis were consistent with these measurements. Thus, the simulation of static loading with our system was validated by the reference data in the literature, suggesting that our FE model of osteocyte-lacunar-canalicular is reliable.

For the cyclic loading simulations, the loading frequency and loading magnitude were investigated in this study, and we performed a total of 20 simulations with different loading strain ad 5 different frequencies (1, 5, 10, 40, and 100 Hz) which are within the physiologically meaningful frequency range of 1–100 Hz [17, 59, 63]. The maximum strain increased with increasing loading frequency and increasing loading magnitude (loading strain). The maximum principal strain occurred at *t*/*T* = 0.5, which matched the maximum value of loading strain. Because the sinusoidal loading was applied in the cyclic loading simulations, the principal strain varied with time. When the loading strain reached the maximum magnitude value, the strain amplification factor of 100 Hz was higher than the other frequencies. When *t* = 0.5*T*, the strain amplification factor increased with loading frequency and with loading strain. However, the strain amplification factor increased only slightly with the increasing loading magnitude for the same loading frequency and increased slightly with the increasing loading frequency for the same loading magnitude.

There has been a lack of experimental data regarding the strain amplification factors for the osteocyte-lacunar-canalicular system. Due to the complex microstructural organization of osteocyte-lacunar-canalicular system, the osteocytes cannot be reliably and easily estimated from global strain measurements [36]. The results of static loading in our study are comparable with FE analysis estimates [14, 22] and digital image measurements [36]. We have to point out that the cyclic loading data of our model is yet to be validated in future studies, as it is beyond the scope of present work. Furthermore, since pore fluid pressure is an important load-induced phenomenon of the osteocyte-lacunar-canalicular system as it in large part dictates the shear stress and degree of chemotransport experienced by the cells, future studies will be required to investigate the pore pressure, flow velocity, and poroelasticity in the 3D poroelastic FE model. Such data will facilitate the understanding of the biomechanical responses to loading and chemotransport around the osteocyte cell body in the osteocyte-lacunar-canalicular system.

Taken together, to understand the changes in strain of osteocytes with different loading frequencies and loading magnitudes, the current study has developed a 3D FE model of osteocyte-lacunar-canalicular system which was used to predict the responses of strain distributions of the osteocyte system under various static and cyclic loads. The study found that the strain amplification factor increased with loading frequency and increasing loading strain.

## Figures and Tables

**Table 1:** Material properties and element information applied in the FE model of osteocyte lacunae.

Components	Young's Module, MPa	Poisson's ratio	Type of element	Number of elements	Reference
ECM	16	0.38	C3D20R	77480	[14]
PCM (with canaliculi)	0.043	0.4	C3D20R	7183	[10]
Osteocyte cell body and processes	0.00447	0.3	C3D20R	23205	[58]

## References

[1] Bonewald L. F. (2011). The amazing osteocyte. *Journal of Bone and Mineral Research*.

[2] Franz-Odendaal T. A., Hall B. K., Witten P. E. (2006). Buried alive: how osteoblasts become osteocytes. *Developmental Dynamics*.

[3] Knothe Tate M. L., Adamson J. R., Tami A. E., Bauer T. W. (2004). The osteocyte. *International Journal of Biochemistry and Cell Biology*.

[4] You L.-D., Weinbaum S., Cowin S. C., Schaffler M. B. (2004). Ultrastructure of the osteocyte process and its pericellular matrix. *Anatomical Record Part A: Discoveries in Molecular, Cellular, and Evolutionary Biology*.

[5] Zhang K., Barragan-Adjemian C., Ye L. (2006). E11/gp38 selective expression in osteocytes: regulation by mechanical strain and role in dendrite elongation. *Molecular and Cellular Biology*.

[6] Bonewald L. F. (2006). Mechanosensation and transduction in osteocytes. *BoneKEy Osteovision*.

[7] Klein-Nulend J., Bakker A. D., Bacabac R. G., Vatsa A., Weinbaum S. (2013). Mechanosensation and transduction in osteocytes. *Bone*.

[8] Terzis J. K., Smith K. J. (1987). Repair of severed peripheral nerves: comparison of the 'de Medinaceli' and standard microsuture methods. *Experimental Neurology*.

[9] Alexopoulos L. G., Setton L. A., Guilak F. (2005). The biomechanical role of the chondrocyte pericellular matrix in articular cartilage. *Acta Biomaterialia*.

[10] Michalek A. J., Iatridis J. C. (2007). A numerical study to determine pericellular matrix modulus and evaluate its effects on the micromechanical environment of chondrocytes. *Journal of Biomechanics*.

[11] Fotia C., Messina G. M. L., Marletta G., Baldini N., Ciapetti G. (2013). Hyaluronan-based pericellular matrix: substrate electrostatic charges and early cell adhesion events. *European Cells and Materials*.

[12] Wang B., Lai X., Price C. (2014). Perlecan-containing pericellular matrix regulates solute transport and mechanosensing within the osteocyte lacunar-canalicular system. *Journal of Bone and Mineral Research*.

[13] Prasadam I., Farnaghi S., Feng J. Q. (2013). Impact of extracellular matrix derived from osteoarthritis subchondral bone osteoblasts on osteocytes: role of integrin*β*1 and focal adhesion kinase signaling cues. *Arthritis Research & Therapy*.

[14] Verbruggen S. W., Vaughan T. J., McNamara L. M. (2012). Strain amplification in bone mechanobiology: a computational investigation of the in vivo mechanics of osteocytes. *Journal of the Royal Society Interface*.

[15] Cowin S. C., Moss-Salentijn L., Moss M. L. (1991). Candidates for the mechanosensory system in bone. *Journal of Biomechanical Engineering*.

[16] Hsieh Y.-F., Turner C. H. (2001). Effects of loading frequency on mechanically induced bone formation. *Journal of Bone and Mineral Research*.

[17] Gururaja S., Kim H. J., Swan C. C., Brand R. A., Lakes R. S. (2005). Modeling deformation-induced fluid flow in cortical bone's canalicular-lacunar system. *Annals of Biomedical Engineering*.

[18] Wang L., Cowin S. C., Weinbaum S., Fritton S. P. (2000). Modeling tracer transport in an osteon under cyclic loading. *Annals of Biomedical Engineering*.

[19] Watchmaker G. P., Gumucio C. A., Crandall R. E., Vannier M. A., Weeks P. M. (1991). Fascicular topography of the median nerve: a computer based study to identify branching patterns. *The Journal of Hand Surgery*.

[20] Sun K., Zhang J., Chen T. (2009). Three-dimensional reconstruction and visualization of the median nerve from serial tissue sections. *Microsurgery*.

[21] McCreadie B. R., Hollister S. J. (1997). Strain concentrations surrounding an ellipsoid model of lacunae and osteocytes. *Computer Methods in Biomechanics and Biomedical Engineering*.

[22] Rath Bonivtch A., Bonewald L. F., Nicolella D. P. (2007). Tissue strain amplification at the osteocyte lacuna: a microstructural finite element analysis. *Journal of Biomechanics*.

[23] Burr D. B., Milgrom C., Fyhrie D. (1996). In vivo measurement of human tibial strains during vigorous activity. *Bone*.

[24] Pazzaglia U. E., Congiu T., Sibilia V., Quacci D. (2014). Osteoblast-osteocyte transformation. A SEM densitometric analysis of endosteal apposition in rabbit femur. *Journal of Anatomy*.

[25] Pazzaglia U. E., Congiu T., Brunelli P. C., Magnano L., Benetti A. (2013). The long bone deformity of osteogenesis imperfecta III: analysis of structural changes carried out with scanning electron microscopic morphometry. *Calcified Tissue International*.

[26] Pazzaglia U. E., Congiu T., Pienazza A., Zakaria M., Gnecchi M., Dell'Orbo C. (2013). Morphometric analysis of osteonal architecture in bones from healthy young human male subjects using scanning electron microscopy. *Journal of Anatomy*.

[27] Marotti G., Muglia M. A., Zaffe D. (1985). A SEM study of osteocyte orientation in alternately structured osteons. *Bone*.

[28] Schneider P., Meier M., Wepf R., Müller R. (2011). Serial FIB/SEM imaging for quantitative 3D assessment of the osteocyte lacuno-canalicular network. *Bone*.

[29] Sharma D., Ciani C., Marin P. A. R., Levy J. D., Doty S. B., Fritton S. P. (2012). Alterations in the osteocyte lacunar-canalicular microenvironment due to estrogen deficiency. *Bone*.

[30] Shah F. A., Johansson B. R., Thomsen P., Palmquist A. (2014). Ultrastructural evaluation of shrinkage artefacts induced by fixatives and embedding resins on osteocyte processes and pericellular space dimensions. *Journal of Biomedical Materials Research Part A*.

[31] Kamioka H., Kameo Y., Imai Y. (2012). Microscale fluid flow analysis in a human osteocyte canaliculus using a realistic high-resolution image-based three-dimensional model. *Integrative Biology*.

[32] Pazzaglia U. E., Congiu T., Marchese M., Zarattini G., Dell'Orbo C. (2012). The canalicular system and the osteoblast domain in human secondary osteons. *Anatomia, Histologia, Embryologia*.

[33] du Plessis L., van Wilpe E. (2009). Thrombocyte morphology and morphometric observations in two vulture species. *Veterinary Clinical Pathology*.

[34] Sugawara Y., Kamioka H., Honjo T., Tezuka K.-I., Takano-Yamamoto T. (2005). Three-dimensional reconstruction of chick calvarial osteocytes and their cell processes using confocal microscopy. *Bone*.

[35] Kamioka H., Honjo T., Takano-Yamamoto T. (2001). A three-dimensional distribution of osteocyte processes revealed by the combination of confocal laser scanning microscopy and differential interference contrast microscopy. *Bone*.

[36] van Hove R. P., Nolte P. A., Vatsa A. (2009). Osteocyte morphology in human tibiae of different bone pathologies with different bone mineral density—is there a role for mechanosensing?. *Bone*.

[37] Vatsa A., Breuls R. G., Semeins C. M., Salmon P. L., Smit T. H., Klein-Nulend J. (2008). Osteocyte morphology in fibula and calvaria—is there a role for mechanosensing?. *Bone*.

[38] Lin Y., Xu S. (2011). AFM analysis of the lacunar-canalicular network in demineralized compact bone. *Journal of Microscopy*.

[39] Ghosh K., Gangodkar S., Jain P. (2008). Imaging the interaction between dengue 2 virus and human blood platelets using atomic force and electron microscopy. *Journal of Electron Microscopy*.

[40] Reilly G. C., Knapp H. F., Stemmer A., Niederer P., Knothe Tate M. L. (2001). Investigation of the morphology of the lacunocanalicular system of cortical bone using atomic force microscopy. *Annals of Biomedical Engineering*.

[41] Dong P., Pacureanu A., Zuluaga M. A. A new quantitative approach for estimating bone cell connections from nano-CT images.

[42] Dong P., Haupert S., Hesse B. (2014). 3D osteocyte lacunar morphometric properties and distributions in human femoral cortical bone using synchrotron radiation micro-CT images. *Bone*.

[43] Nango N., Kubota S., Takeuchi A. (2013). Talbot-defocus multiscan tomography using the synchrotron X-ray microscope to study the lacuno-canalicular network in mouse bone. *Biomedical Optics Express*.

[44] Andrews J. C., Almeida E., van der Meulen M. C. (2010). Nanoscale X-ray microscopic imaging of mammalian mineralized tissue. *Microscopy & Microanalysis*.

[45] Langer M., Pacureanu A., Suhonen H., Grimal Q., Cloetens P., Peyrin F. (2012). X-ray phase nanotomography resolves the 3D human bone ultrastructure. *PLoS ONE*.

[46] Verbruggen S. W., Vaughan T. J., McNamara L. M. (2014). Fluid flow in the osteocyte mechanical environment: a fluid-structure interaction approach. *Biomechanics and Modeling in Mechanobiology*.

[47] Deligianni D. D., Apostolopoulos C. A. (2008). Multilevel finite element modeling for the prediction of local cellular deformation in bone. *Biomechanics and Modeling in Mechanobiology*.

[48] Jabaley M. E., Wallace W. H., Heckler F. R. (1980). Internal topography of major nerves of the forearm and hand: a current view. *Journal of Hand Surgery*.

[49] Steck R., Niederer P., Knothe Tate M. L. (2003). A finite element analysis for the prediction of load-induced fluid flow and mechanochemical transduction in bone. *Journal of Theoretical Biology*.

[50] Beno T., Yoon Y.-J., Cowin S. C., Fritton S. P. (2006). Estimation of bone permeability using accurate microstructural measurements. *Journal of Biomechanics*.

[51] McNamara L. M., Majeska R. J., Weinbaum S., Friedrich V., Schaffler M. B. (2009). Attachment of osteocyte cell processes to the bone matrix. *Anatomical Record*.

[52] Remaggi F., Canè V., Palumbo C., Ferretti M. (1998). Histomorphometric study on the osteocyte lacuno-canalicular network in animals of different species. I. Woven-fibered and parallel-fibered bones. *Italian Journal of Anatomy and Embryology*.

[53] Ferretti M., Muglia M. A., Remaggi F., Canè V., Palumbo C. (1999). Histomorphometric study on the osteocyte lacuno-canalicular network in animals of different species. II. Parallel-fibered and lamellar bones. *Italian Journal of Anatomy and Embryology*.

[54] Pazzaglia U. E., Congiu T. (2013). The cast imaging of the osteon lacunar-canalicular system and the implications with functional models of intracanalicular flow. *Journal of Anatomy*.

[55] Wang L., Wang Y., Han Y. (2005). In situ measurement of solute transport in the bone lacunar-canalicular system. *Proceedings of the National Academy of Sciences of the United States of America*.

[56] Tsouknidas A., Savvakis S., Asaniotis Y., Anagnostidis K., Lontos A., Michailidis N. (2013). The effect of kyphoplasty parameters on the dynamic load transfer within the lumbar spine considering the response of a bio-realistic spine segment. *Clinical Biomechanics*.

[57] Alexopoulos L. G., Haider M. A., Vail T. P., Guilak F. (2003). Alterations in the mechanical properties of the human chondrocyte pericellular matrix with osteoarthritis. *Journal of Biomechanical Engineering*.

[58] Sugawara Y., Ando R., Kamioka H. (2008). The alteration of a mechanical property of bone cells during the process of changing from osteoblasts to osteocytes. *Bone*.

[59] Goulet G. C., Coombe D., Martinuzzi R. J., Zernicke R. F. (2009). Poroelastic evaluation of fluid movement through the lacunocanalicular system. *Annals of Biomedical Engineering*.

[60] Turner C. H., Forwood M. R., Otter M. W. (1994). Mechanotransduction in bone: Do bone cells act as sensors of fluid flow?. *FASEB Journal*.

[61] Nicolella D. P., Lankford J. (2002). Microstructural strain near osteocyte lacuna in cortical bone in vitro. *Journal of Musculoskeletal Neuronal Interactions*.

[62] Nicolella D. P., Moravits D. E., Gale A. M., Bonewald L. F., Lankford J. (2006). Osteocyte lacunae tissue strain in cortical bone. *Journal of Biomechanics*.

[63] Fritton S. P., McLeod K. J., Rubin C. T. (2000). Quantifying the strain history of bone: spatial uniformity and self-similarity of low-magnitude strains. *Journal of Biomechanics*.

